# Comparing the Clinical and Neuropsychological Characteristics of Parkinson’s Disease With and Without Freezing of Gait

**DOI:** 10.3389/fnins.2021.660340

**Published:** 2021-04-27

**Authors:** Reyisha Taximaimaiti, Xiao-Ping Wang

**Affiliations:** Department of Neurology, Shanghai TongRen Hospital, Shanghai Jiao Tong University School of Medicine, Shanghai, China

**Keywords:** Parkinson’s disease, freezing of gait, neuropsychological assessment, cognitive impairment, striatum

## Abstract

**Introduction:**

Freezing of gait (FOG) is one of the most common walking problems in Parkinson’s disease (PD). Impaired cognitive function is believed to play an important role in developing and aggravating FOG in PD. But some evidence suggests that motor function discrepancy may affect testing results. Therefore, we think it is necessary for PD-FOG(+) and PD-FOG(−) patients to complete neuropsychological tests under similar motor conditions.

**Methods:**

This study recruited 44 idiopathic PD patients [PD-FOG(+) *n* = 22, PD-FOG(−) *n* = 22] and 20 age-matched healthy controls (HC). PD-FOG(+) and PD-FOG(−) patients were matched for age, year of education, and Hoehn and Yahr score (H&Y). All participants underwent a comprehensive battery of neuropsychological assessment, and demographical and clinical information was also collected.

**Results:**

PD patients showed poorer cognitive function, higher risks of depression and anxiety, and more neuropsychiatric symptoms compared with HC. When controlling for age, years of education, and H&Y, there were no statistical differences in cognitive function between PD-FOG(+) and PD-FOG(−) patients. But PD-FOG(+) patients had worse motor and non-motor symptoms than PD-FOG(−) patients. PD patients whose motor symptoms initiated with rigidity and initiated unilaterally were more likely to experience FOG.

**Conclusion:**

Traditional neuropsychological testing may not be sensitive enough to detect cognitive impairment in PD. Motor symptoms initiated with rigidity and initiated unilaterally might be an important predictor of FOG.

## Introduction

Freezing of gait (FOG) is described as brief and episodic forward stepping obstacles while walking and initiating (Nutt et al., 2011). It is often reported as feeling like “feet glued to the floor” and can be triggered or exacerbated by crossing narrow aisles, turning around, and doing dual tasks (Piramide et al., 2020). FOG is one of the most common walking problems in advanced Parkinson’s disease (PD) (Ortelli et al., 2019). It not only increases patients’ risk of falls and injuries, but also leads to emotional disorders and poor quality of life (Morrist et al., 2020; Rahimpour et al., 2020). Although some pharmacological and physical interventions are used to alleviate FOG in PD patients, the results are still far from satisfactory (Onder and Ozyurek, 2020).

The underlying pathophysiology of FOG is not fully understood (Vitorio et al., 2020). It is widely recognized that cognitive impairment (CI) plays an important role in developing and aggravating FOG in PD. Many studies suggest that FOG is associated with decline in a variety of neuropsychological domains, including executive function, attention, memory, language, and visuospatial function, and CI can, in turn, aggravate FOG (Onder and Ozyurek, 2020). But, in most of these studies, motor symptoms of PD-FOG(+) patients are significantly more severe than that of PD-FOG(−) patients, which puts PD-FOG(+) patients at a natural disadvantage. What is more, a recent study involving 227 participants suggests that the severity of FOG may not synchronize with the severity of CI, indicating that motor function discrepancy may affect the results of neuropsychological tests (Morrist et al., 2020). Functional imaging studies also found that the mechanism of FOG is related to advanced cognitive and attention-related networks rather than motor networks (Li Y. et al., 2020). Therefore, we think it is necessary for PD-FOG(+) and PD-FOG(−) patients to complete neuropsychological tests under similar motor conditions to reduce the impact of motor function discrepancy on the results. In addition, previous studies demonstrate that FOG in PD patients is also associated with anxiety, depression, poor sleep quality, higher levodopa equivalent dose (LED), worse balance, and more motor/non-motor complications (de Almeida et al., 2021; Lichter et al., 2021). Whether these factors change as the motor function discrepancy decreases is also worth exploring.

In this study, we conducted detailed neuropsychological assessments on PD-FOG(+) and PD-FOG(−) patients whose age, years of education, and Hoehn and Yahr score (H&Y) were matched to explore whether FOG in PD was associated with impairment in certain neuropsychological domains. We also collected their demographical and clinical information to find characteristics correlated with FOG. We hypothesized that PD-FOG(+) patients would have poorer cognitive function and more severe clinical symptoms than PD-FOG(−) patients.

## Materials and Methods

### Participants

This study included 44 idiopathic PD patients and 20 age-matched non-PD healthy controls (HC) from May 1 to December 31, 2020. All participants were recruited to this study at the outpatient department of Shanghai TongRen Hospital. PD diagnoses were specified by a neurologist at Shanghai TongRen Hospital. PD-FOG(+) and PD-FOG(−) patients recruited in this study were matched for age, years of education, and H&Y.

The enrolled age of PD patients and HC was between 50 and 85 years old. The inclusion criteria for PD patients were as follows: (1) met the diagnosis of idiopathic PD according to UK Brain Bank Criteria (Hughes et al., 1992), (2) score on the Montreal Cognitive Assessment (MoCA) ≥ 18, and (3) agreed to participate in this study. The exclusion criteria were as follows: (1) serious emotional disorders or psychiatric disorders, such as depression, anxiety, schizophrenia, mania, etc.; (2) serious cognitive dysfunctions, such as Alzheimer’s disease, vascular dementia, frontotemporal dementia, etc.; (3) history of other neurological disease, such as progressive superanuclear palsy, multiple system atrophy, multiple sclerosis, etc.; (4) history of severe brain injury; (5) history of cancer or brain tumor; (6) serious systemic disease, such as Hashimoto’s encephalopathy, systemic lupus erythematosus, acquired immune deficiency syndrome, etc.; or (7) obvious visual or auditory defects. Informed consent was gained from all participants. This study was approved by the Medical Ethics Committee of Shanghai TongRen Hospital (ID: 2020-084-01).

### Demographic Characteristics and Clinical Symptoms

Basic information was collected from all participants including age, sex, years of education, age at PD onset, PD duration, and body mass index [BMI, = weight (in kg)/height^2^ (in m)]. Total daily antiparkinsonian drugs were estimated as LED.

Motor symptom severity was assessed using part III of the Movement Disorders Society Unified Parkinson’s disease rating scale (MDS-UPDRS-III) and H&Y. PD participants were then classified as PD-FOG(+) if they answered “yes” to the third question of the Freezing of Gait Questionnaire (FOG-Q) (“Feel like your feet are sticking to the floor while you are walking, turning, or trying to walk?”) and classified as PD-FOG(−) if their answer was “no.” Balance function was assessed via the Tinetti mobility test, which included balance gait scores. Activities of daily living (ADL) involved basic ADL (eight items) and instrumental ADL (12 items). The Hamilton Depression Scale (HAMD) and Hamilton Anxiety Scale (HAMA) were used to measure depression and anxiety symptoms, respectively. The Neuropsychiatric Inventory Questionnaire (NPI-Q) was used to evaluate participants’ neuropsychiatric symptoms. All PD patients were assessed in the practical “On” medication state.

Clinical symptoms were obtained through clinical observation, relevant questionnaire results, and medical history collection. Specifically, initial motor symptoms were obtained via tracing participants’ past medical records and reconfirmed by the patients themselves. Motor symptoms included typical PD motor symptoms and common motor complications, which were obtained through physical examination and medical history (Jankovic, 2008). Non-motor symptoms included dysautonomia, emotional disorders, sensory symptoms, and sleep disorders, which were obtained through medical history, the Non-Motor Symptoms Scale (NMSS), and the Parkinson’s Disease Sleep Scale (PDSS) (only answers were “yes” or “no” regardless of the degree). It should be emphasized that those PD patients who complained of anxiety/depression did not meet the clinical diagnostic criteria of anxiety/depression after the HAMA and HAMD tests. All patients were assessed in the “On” state.

### Neuropsychological Assessment

All participants underwent a comprehensive battery of neuropsychological tests, evaluating global cognition, memory function, executive function, language function, attention and working memory, visuospatial ability, and emotion recognition ability (Xu et al., 2018). These tests were conducted by a trained neurology MD.

Global cognition was assessed by the Mini-Mental State Examination (MMSE) and MoCA. Memory function was assessed by the Auditory Verbal Learning Test (AVLT) and the Brief Visuospatial Memory Test–Revised (BVMT-R). Executive function was assessed by the Stroop Color−Word Test (SCWT), Trail Making Test (TMT) Part B, and the time of TMT Part B minus Part A (TMT B-A). Language function was assessed by the Boston Naming Test and Verbal Fluency Test (VFT). Attention and working memory were assessed by the Symbol Digit Modalities Test (SDMT), Digital Span Test (DST), and TMT Part A. Visuospatial ability was assessed using the Judgment of Line Orientation (JLO) and Clock Drawing test (CDT). The Eye Expression Recognition Test (EERT) was used to investigate participants’ emotional recognition ability by recognizing simple and complex human mental states from eye expressions alone (Kington et al., 2000).

### Statistical Analysis

SPSS software version 24.0 (IBM, Chicago, IL, United States) was used to evaluate all the data in this study. Numerical variables were described as mean ± standard deviation. Categorical variables were described as frequencies and percentages. Independent samples *t*-tests and chi-square tests were used to compare differences in demographic characteristics and neuropsychological assessment between PD and HC and PD-FOG(+) and PD-FOG(−) when appropriate. Differences in clinical characteristics were compared using chi-square tests between PD-FOG(+) and PD-FOG(−). A two-tailed *P*-value less than 0.05 was considered statistically significant.

## Results

### Demographic Characteristics and Clinical Symptoms Results

The results on the demographic characteristics of participants are presented in Table 1. This study recruited 44 PD patients and 20 HC. Of those with PD, 22 were classified as PD-FOG(+) and 22 were classified as PD-FOG(−). When comparing PD patients with HC, two groups showed no differences in age, years of education, and BMI. However, PD patients were more likely to have worse daily activities behaviors and neuropsychiatric symptoms (*P* < 0.001) as well as more chances to suffer from depression and anxiety (*P* < 0.001).

**TABLE 1:** Demographical characteristics and clinical symptoms of PD, PD-FOG(+), PD-FOG(−) patients, and HC.

	**PD (*n* = 44)**	**PD-FOG(+) (*n* = 22)**	**PD-FOG(−) (*n* = 22)**	**HC (*n* = 20)**	**Independent *T*-test**	
					**PD vs. HC**	**PD-FOG(+) vs. PD-FOG(−)**
Age (years)	69.2 ± 7.1	67.7 ± 7.3	70.7 ± 6.7	68.2 ± 6.8	0.61	0.17
Sex (m/f)*	37/7	21/1	16/6	13/7	**0.04**	0.09
Education (years)	12.1 ± 3.2	11.7 ± 3.7	12.6 ± 2.6	11.6 ± 2.4	0.41	0.35
Age at PD onset (years)	62.5 ± 8.4	59.9 ± 7.0	65.2 ± 9.0	–	–	**0.03**
Disease duration (years)	6.8 ± 4.9	7.9 ± 4.7	5.6 ± 4.8	–	–	0.11
BMI	24.4 ± 2.6	25.1 ± 1.9	23.7 ± 3.0	23.8 ± 2.4	0.33	0.06
LED (mg)	465.2 ± 276.9	562.5 ± 328.9	367.9 ± 170.3	–	–	**0.02**
UPDRS-III	15.5 ± 7.4	17.0 ± 7.3	14.0 ± 7.2	–	–	0.17
H&Y (1/2/3/4) *	18/11/12/3	7/6/8/1	11/5/4/2	–	–	0.45
FOG-Q	7.3 ± 5.3	12.0 ± 3.2	2.6 ± 1.3	–	–	**<0.001**
Total Tinetti score	23.8 ± 4.1	21.9 ± 3.8	25.7 ± 3.6	–	–	**<0.01**
Tinetti balance score	13.2 ± 2.6	12.2 ± 2.4	14.3 ± 2.5	–	–	**<0.01**
Tinetti gait score	10.6 ± 1.8	9.7 ± 1.7	11.5 ± 1.4	–	–	**<0.01**
ADL	24.3 ± 5.3	25.2 ± 6.8	23.3 ± 3.0	20.5 ± 0.7	**<0.001**	0.23
HAMA	11.8 ± 6.9	15.2 ± 5.9	8.4 ± 6.2	5.0 ± 3.4	**<0.001**	**<0.001**
HAMD	9.0 ± 6.5	12.3 ± 5.2	5.7 ± 6.0	3.2 ± 2.5	**<0.001**	**<0.001**
NPI-Q	25.9 ± 14.5	20.9 ± 16.3	10.9 ± 10.6	3.9 ± 4.5	**<0.001**	**0.02**
**Initial motor symptoms**						
Tremor (n,%)*	17, 38.6%	5, 22.7%	12, 54.5%	–	–	**0.03**
Bradykinesia (n,%)*	17, 38.6%	9, 40.9%	8, 36.4%	–	–	0.76
Rigidity (n,%)*	20, 45.5%	14, 63.6%	6, 27.3%	–	–	**0.02**
Unilateral disease (n,%)*	29, 65.9%	18, 81.8%	11, 50.0%	–	–	**0.03**
Bilateral disease (n,%)*	15, 34.1%	4, 18.2%	11, 50.0%	–	–	**0.03**
**Motor symptoms at exam**						
Tremor (n,%)*	33, 75.0%	16, 72.7%	17, 77.3%	–	–	0.73
Bradykinesia (n,%)*	41, 93.2%	21, 95.5%	20, 90.9%	–	–	0.55
Rigidity (n,%)*	36, 81.8%	20, 90.9%	16, 72.7%	–	–	0.12
Unilateral disease (n,%)*	15, 34.1%	8, 36.4%	7, 31.8%	–	–	0.75
Bilateral disease (n,%)*	29, 65.9%	14, 63.6%	15, 68.2%	–	–	0.75
Postural abnormality (crooked, unstable) (n,%)*	31, 70.5%	18, 81.8%	13, 59.1%	–	–	0.10
Dyskinesia (n,%)*	6, 13.6%	5, 22.7%	1, 4.5%	–	–	0.09
Motor fluctuation (n,%)*	16, 36.4%	12, 54.5%	4, 18.2%	–	–	**0.01**
Fall (n,%)*	20, 45.5%	16, 72.7%	4, 18.2%	–	–	**<0.001**
Difficulty turning around (n,%)*	28, 63.6%	18, 81.8%	10, 45.5%	–	–	**0.01**
Cramps (limbs, face) (n,%)*	20, 45.5%	11, 50.0%	9, 40.9%	–	–	0.55
Step reduction (n,%)*	22, 50.0%	13, 59.1%	9, 40.9%	–	–	0.23
Blepharospasm (n,%)*	14, 31.8%	8, 36.4%	6, 27.3%	–	–	0.52
**Non-motor symptoms at exam**						
Hyposmia (n,%)*	8, 18.2%	5, 22.7%	3, 13.6%	–	–	0.43
Orthostatic hypotension (n,%)*	14, 31.8%	8, 36.4%	6, 27.3%	–	–	0.52
Constipation (n,%)*	34, 77.3%	18, 81.8%	16, 72.7%	6, 30.0%	**<0.001**	0.47
Urinary dysfunction (n,%)*	20, 45.5%	12, 54.5%	8, 36.4%	2, 10.0%	**<0.01**	0.23
Weight loss (n,%)*	26, 59.1%	17, 77.3%	9, 40.9%	–	–	**0.01**
Fatigue (n,%)*	27, 61.4%	16, 72.7%	11, 50.0%	–	–	0.12
Paresthesia (n,%)*	20, 45.5%	12, 54.5%	8, 36.4%	1, 5.0%	**0.001**	0.23
Pain (n,%)*	17, 38.6%	11, 50.0%	6, 27.3%	4, 20.0%	0.14	0.12
Anosmia (n,%)*	5, 11.4%	3, 13.6%	2, 9.1%	0, 0.0%	0.12	0.64
Apathy (n,%)*	15, 34.1%	9, 40.9%	6, 27.3%	0, 0.0%	**<0.01**	0.34
Anhedonia (n,%)*	11, 25.0%	7, 31.8%	4, 18.2%	1, 5.0%	0.06	0.30
Depression (n,%)*	7, 15.9%	5, 22.7%	2, 9.1%	0, 0.0%	0.06	0.22
Anxiety (n,%)*	17, 38.6%	13, 59.1%	4, 18.2%	1, 5.0%	**<0.01**	**<0.01**
Hallucination (n,%)*	19, 43.2%	11, 50.0%	8, 36.4%	0, 0.0%	**<0.001**	0.36
Delusion (n,%)*	8, 18.2%	6, 27.3%	2, 9.1%	0, 0.0%	**0.04**	0.12
Impulse control disorder (n,%)*	19, 43.2%	14, 63.6%	5, 22.7%	0, 0.0%	**<0.001**	**<0.01**
Insomnia (n,%)*	22, 50.0%	15, 68.2%	7, 31.8%	4, 20.0%	**0.02**	**0.02**
RBD (n,%)*	33, 75.0%	17, 77.3%	16, 72.7%	0, 0.0%	**<0.001**	0.73
RLS (n,%)*	7, 15.9%	5, 22.7%	2, 9.1%	0, 0.0%	0.06	0.22

BMI, body mass index; LED, levodopa equivalent dose; MDS-UPDRS-III, part III of the Movement Disorders Society Unified Parkinson’s disease rating scale; H & Y, Hoehn and Yahr score; FOG-Q, Freezing of Gait Questionnaire; ADL, Activities of Daily Living; HAMA, Hamilton Anxiety Scale; HAMD, Hamilton Depression Scale; NPI-Q, Neuropsychiatric Inventory Questionnaire; RBD, REM behavior disorder; RLS, Restless leg syndrome. *Chi-squared test. Bold values means emphasize results p < 0.05, which we set as having statistical differences.

According to our results, the age at PD onset of PD-FOG(+) patients was younger than PD-FOG(−) patients (*P* = 0.03). PD-FOG(+) patients also took higher daily LED than PD-FOG(−) patients (*P* = 0.02). PD-FOG(+) patients performed worse in the FOG-Q (*P* < 0.001) and Tinetti mobility test, no matter their balance or gait (*P* < 0.01). NPI-Q, HAMA, and HAMD showed that PD-FOG(+) patients were more likely to have neuropsychiatric symptoms (*P* = 0.02), anxiety (*P* < 0.001), and depression (*P* < 0.001) than PD-FOG(−) patients. No differences were seen in age, sex, years of education, PD duration, BMI, UPDRS-III score, H&Y, and ADL between the two subgroups.

In Table 1, we also display the differences of clinical characteristics. PD-FOG(+) and PD-FOG(−) patients showed significant differences in initial motor symptoms. PD-FOG(−) patients tended to have a bilateral (*P* = 0.03) and tremor (*P* = 0.03) onset, and PD-FOG(+) patients tended to have a unilateral (*P* = 0.03) and rigid (*P* = 0.02) onset. As for motor symptoms at exam, PD-FOG(+) patients had more chances to experience motor fluctuation (*P* = 0.01), falls (*P* < 0.001), and difficulty turning around (*P* = 0.01). For non-motor symptoms, PD-FOG(+) patients tended to have more chances to suffer from weight loss (*P* = 0.01), anxiety (*P* < 0.01), impulse control disorder (*P* < 0.01), and insomnia (*P* = 0.02).

### Neuropsychological Assessment Results

Table 2 shows the results of the neuropsychological assessment. PD patients had worse performances than the HC group in global cognition tests and almost all batteries of neuropsychological tests except for AVLT-immediate recall-2nd time (*P* = 0.06), SDMT (*P* = 0.05), DST backward (*P* = 0.13), JLO (*P* = 0.54), and CDT (*P* = 0.12). But no differences were seen between PD-FOG(+) patients and PD-FOG(−) patients in global cognition tests and the six batteries of neuropsychological tests (*P* > 0.05).

**TABLE 2:** Neuropsychological assessment of PD, PD-FOG(+), PD-FOG(−) patients, and HC.

	**PD (*n* = 44)**	**PD-FOG(+) (*n* = 22)**	**PD-FOG(−) (*n* = 22)**	**HC (*n* = 20)**	**Independent *T*-test**	
					**PD vs. HC**	**PD-FOG(+) vs. PD-FOG(−)**
**Global cognition**						
MMSE	27.3 ± 1.9	27.3 ± 1.8	27.3 ± 2.0	28.5 ± 1.5	**<0.01**	0.94
MoCA	25.3 ± 3.2	25.3 ± 2.9	25.4 ± 3.6	27.6 ± 1.5	**<0.001**	0.89
**Memory function**						
AVLT						
Immediate recall-1st time	3.1 ± 1.5	3.1 ± 1.7	3.0 ± 1.4	4.2 ± 2.2	**<0.05**	0.77
Immediate recall-2nd time	5.2 ± 1.9	5.3 ± 2.1	5.2 ± 1.7	7.3 ± 2.2	**<0.01**	0.88
Immediate recall-3rd time	6.4 ± 2.0	6.3 ± 2.1	6.5 ± 1.9	8.9 ± 2.0	**<0.001**	0.65
5−min delayed recall	4.6 ± 2.4	4.6 ± 2.8	4.5 ± 2.0	8.2 ± 2.4	**<0.001**	0.95
20−min delayed recall	4.5 ± 2.7	4.5 ± 3.1	4.5 ± 2.3	7.9 ± 2.2	**<0.001**	1.00
Cued recall	4.5 ± 2.8	4.5 ± 3.0	4.5 ± 2.5	8.2 ± 2.6	**<0.001**	0.96
Recognition	20.0 ± 2.9	20.1 ± 3.1	20.0 ± 2.8	22.1 ± 1.9	**<0.01**	0.84
BVMT-R						
Immediate recall-1st time	3.2 ± 1.7	3.1 ± 1.3	3.2 ± 2.0	4.1 ± 1.5	**0.04**	0.86
Immediate recall-2nd time	6.9 ± 2.8	7.0 ± 3.0	6.8 ± 2.6	8.1 ± 2.1	0.06	0.83
Immediate recall-3rd time	8.0 ± 2.8	7.8 ± 3.0	8.2 ± 2.6	9.6 ± 2.1	**0.02**	0.63
5−min delayed recall	7.6 ± 3.0	7.6 ± 3.1	7.6 ± 3.0	9.5 ± 2.3	**0.01**	0.96
20−min delayed recall	7.5 ± 3.1	7.7 ± 3.1	7.4 ± 3.2	9.3 ± 2.3	**0.01**	0.71
Recognition	10.2 ± 2.7	10.4 ± 2.6	10.0 ± 2.8	11.7 ± 1.0	**<0.01**	0.54
**Executive function**						
TMT-B time (s)	160.5 ± 58.6	173.4 ± 60.3	147.5 ± 55.3	117.0 ± 25.0	**<0.001**	0.15
TMT-B error responses	4.5 ± 4.8	5.0 ± 5.7	4.1 ± 3.8	2.0 ± 1.6	**<0.01**	0.56
TMT B-A (s)	92.5 ± 37.7	96.2 ± 33.1	88.7 ± 42.3	69.5 ± 22.0	**<0.01**	0.52
SCWT time (s)	40.8 ± 10.3	42.8 ± 11.4	38.8 ± 8.9	33.5 ± 8.3	**<0.01**	0.19
SCWT correct responses	21.4 ± 2.7	21.1 ± 3.3	21.7 ± 2.1	22.8 ± 1.7	**0.02**	0.52
**Language function**						
Boston Naming Test	26.4 ± 2.8	26.8 ± 3.1	26.0 ± 2.6	27.7 ± 1.6	**0.03**	0.35
VFT−fruits	11.1 ± 2.4	11.0 ± 2.5	11.3 ± 2.5	12.9 ± 2.1	**<0.01**	0.63
VFT−animals	17.4 ± 3.6	17.8 ± 3.7	17.0 ± 3.7	22.2 ± 5.3	**<0.01**	0.46
VFT−fruits and animals	12.8 ± 2.9	12.9 ± 3.3	12.6 ± 2.6	15.5 ± 2.4	**<0.001**	0.80
**Attention and working memory**						
SDMT	32.3 ± 9.8	33.6 ± 9.7	30.9 ± 10.0	38.2 ± 11.1	0.05	0.36
DST forward	10.5 ± 2.2	10.8 ± 2.3	10.1 ± 2.1	11.6 ± 1.9	**<0.05**	0.34
DST backward	6.4 ± 1.9	6.6 ± 2.2	6.2 ± 1.5	7.1 ± 1.6	0.13	0.48
DST forward + backward	16.8 ± 3.5	17.4 ± 4.1	16.3 ± 2.9	18.7 ± 2.7	**0.03**	0.33
TMT-A time (s)	69.1 ± 37.9	77.2 ± 48.8	66.1 ± 20.6	47.5 ± 8.8	**<0.01**	0.17
TMT-A error responses	1.2 ± 1.9	1.5 ± 2.4	0.7 ± 1.1	0.5 ± 0.8	**0.03**	0.27
**Visuospatial ability**						
JLO	22.7 ± 4.0	22.4 ± 3.7	23.0 ± 4.2	23.5 ± 5.1	0.54	0.63
CDT	10.9 ± 1.4	10.7 ± 1.8	11.1 ± 0.8	11.4 ± 0.7	0.12	0.33
**Emotion recognition ability**						
EERT	16.9 ± 4.6	16.1 ± 3.7	17.6 ± 5.3	20.9 ± 4.1	**<0.01**	0.30

**MMSE, Mini-Mental State Examination; MoCA, Montreal Cognitive Assessment; AVLT, Auditory Verbal Learning Test; BVMT-R, Brief Visuospatial Memory Test-Revised; TMT, Trail Making Test; SCWT, Stroop Color−Word Test; VFT, Verbal Fluency Test; SDMT, Symbol Digit Modalities Test; DST, Digital Span Test; JLO, Judgment of Line Orientation; CDT, Clock Drawing Test; EERT, Eye Expression Recognition Test.* Bold values means emphasize results p < 0.05, which we set as having statistical differences.*

## Discussion

In this study, we investigated demographical, neuropsychological, and clinical differences between HC and PD, with and without FOG, who were matched for age, years of education, and H&Y. When comparing PD patients with the HC group, PD patients showed poorer cognitive function, higher risks of depression and anxiety, and more neuropsychiatric symptoms. These results were consistent with general clinical observations and previous studies (Morrist et al., 2020). When comparing PD-FOG(+) patients with PD-FOG(−) patients, no differences were seen in cognitive function, but significant differences could be seen in neuropsychiatric and clinical symptoms. According to our results, risk factors for FOG included younger age at PD onset, higher daily LED, and higher NPI; motor symptoms included motor fluctuation, falls, and difficulty turning around; and non-motor symptoms included weight loss, impulse control disorder, depression, anxiety, and insomnia. PD patients whose motor symptoms initiated with rigidity and initiated unilaterally were more likely to experience FOG.

In our study, age, years of education, and H&Y were matched for PD-FOG(+) and PD-FOG(−) patients. Although PD-FOG(+) patients had additional FOG problems, the scores of UPDRS-III in the two subgroups had no significant differences (*P* = 0.17), and all tests were done in the “On” stage, ensuring motor function discrepancy had as little impact as possible on the results of the tests. Under such conditions, neuropsychological assessment showed no statistical differences in cognitive function between PD-FOG(+) and PD-FOG(−) patients. This result was different from our consensus on the relationship between FOG and CI. The reasons might be related to the following points.

First, some cognitive differences may be caused by motor function discrepancy. In most studies, the PD-FOG(+) group has significantly higher UPDRS-III scores than the PD-FOG(−) group. More severe motor symptoms put PD-FOG(+) patients at a natural disadvantage, so it is not surprising that the test results are bound to be more unfavorable for them. For example, severe rigidity and bradykinesia of hands can slow down a patient’s drawing (e.g., Trail Making Test) and writing (e.g., Symbol Digit Modalities Test) speed and result in poor performance in executive function and attention (Wang et al., 2017). Similarly, throat muscle spasms can cause slow and inaudible speech, decreasing the number of objects in the Verbal Fluency Test and eventually reflected as low language ability (Rosenthal et al., 2017; Smith and Caplan, 2018). At the same time, neuroimaging shows that the lesion location and the abnormality of the brain network connection of FOG seems to be different from that of other motor dysfunctions in PD patients. For example, in structural MRI, declined motor functions/higher UPDRS-III score are related to cortical thinning in the caudate, fusiform gyrus, and left temporal pole although FOG is more associated with cortical atrophy in the mesial frontal and cingulate cortices (Zarei et al., 2013; Vastik et al., 2017; Gao et al., 2018). In resting-state functional MRI, PD patients’ motor symptoms, such as tremor and postural instability, are associated with altered regional homogeneity (ReHo) in the precentral gyrus, supplementary motor area (SMA), and cerebellum although the mechanism of FOG seems to be related to advanced cognitive and attention-related networks rather than motor networks (Wang et al., 2019; Li Y. et al., 2020; Li J.-Y. et al., 2020). Therefore, when studying FOG, if the PD-FOG (+) and PD-FOG(−) subgroups can complete neuropsychological tests under similar motor symptoms to minimize the interference of other motor symptoms on the results, it may be more conducive to finding related factors. In fact, even within the PD-FOG(+) group, the occurrence of FOG could range from multiple times a day to once in several months, which divides FOG into mild, moderate, and severe types. This severity discrepancy of FOG is enough to cause a series of differences in clinical symptoms, emotional condition, and brain network activity (Martens et al., 2016; Piramide et al., 2020), not to mention that, when comparing the PD-FOG(+) and PD-FOG(−) patients, if the discrepancy of motor function is too large, there will only be more factors interfering with the test results. However, the association between FOG severity and cognitive function is still full of controversy with some studies finding FOG severity and cognitive function to be negatively correlated and others finding no association between them (Yao et al., 2017; Morrist et al., 2020). Therefore, we thought it was necessary to control motor function discrepancy in the PD-FOG(+) and PD-FOG(−) subgroups.

Second, traditional neuropsychological testing may not be sensitive enough to detect CI in PD. Commonly used cognitive function detection methods mostly come from studies on Alzheimer’s disease (AD) and other types of dementia, which are very suitable to detect cortical dysfunction (Sitek et al., 2015). However, CI of PD may be more complicated than that. On one hand, the typical pathophysiology of PD is loss of dopaminergic neurons within the substantia nigra pars compacta (SNc) and dopamine depletion in the striatum (Mullin and Schapira, 2015). Thus, α-synuclein/Lewy pathology in PD could not only be seen in the striatum, but also in the cortex, brainstem, and limbic system (Alecu and Bennett, 2019). Results of the serial reaction time task (SRTT) and the mirror reading task show that PD patients have abnormal procedural learning and striatum dysfunction, but whether it is related to the formation of FOG is still controversial (Panouilleres et al., 2016; Clark and Lum, 2017). On the other hand, PD patients also have progressive cortical thinning and volume loss in the long run, which is reflected as declines in traditional neuropsychological tests (Filippi et al., 2020). Pathological studies have also shown that PD and AD share a certain degree of neuropathological overlap, and AD neuropathologic change contributes to CI in PD (Colloby et al., 2020; Tong and Chen, 2021). These factors make it difficult to clearly distinguish the CI caused by PD only from the CI caused by PD plus AD (Smith et al., 2019). Evidence also shows that PD patients with FOG are more likely to develop CI and even dementia than those without FOG, indicating FOG may have a unique neural circuit abnormality or pathology (Factor et al., 2014; Palermo et al., 2019). Fortunately, neuroimaging technology gives us a new option to learn FOG. Structural and functional neuroimaging studies show that FOG in PD may relate to a widespread structural and functional impairment in cortical and subcortical brain structures (Bharti et al., 2019). Functional neuroimaging also makes it possible to combine clinical symptom and neuropsychological assessment results with a specific neural network or cortical area. For example, diminished spatial and verbal working memory skills are assumed to be caused by dopaminergic dysfunction in the frontostriatal circuit (Wojtala et al., 2019). Executive dysfunction and FOG seems to share atrophic neurodegenerative changes in the same cortical area (Brugger et al., 2015). Neuroimaging combined with more targeted neuropsychological tests for striatal function might be a future direction.

In addition, the results of neuropsychological tests can be affected by many other factors, such as age, sex, mood, attention level, and familiarity (Sturm, 2007; Hoijer et al., 2020). A high level of LED can also increase the risk of FOG dramatically (Vercruysse et al., 2012). Therefore, how to eliminate or control the influence of interfering factors when we study FOG remains a challenge.

The results of clinical symptom assessment drew a worse picture of motor and non-motor symptoms for PD-FOG(+) patients. According to our results, PD patients whose motor symptoms initiated with rigidity and initiated unilaterally were more likely to experience FOG. This may link to PD’s characteristically asymmetric onset of motor symptoms with which the initially affected side remains most prominently affected even though the contralateral side becomes involved later on (Boonstra et al., 2014; Prasad et al., 2018). Unilateral onset, especially the unilateral onset of legs, can lead to disrupted stepping and asymmetric gait/balance, which could then lead to FOG (Plotnik et al., 2008; Boonstra et al., 2014). Rigidity, rather than tremor or bradykinesia, can exacerbate this kind of asymmetry in gait/balance and eventually increase the risk of FOG (Bartels et al., 2003; Kwon et al., 2014). Some studies also suggest that rigid-dominant PD patients have worse cognitive function compared with tremor-dominant PD patients (Wojtala et al., 2019). They think these differences are caused because rigid-dominant patients suffer from deficits in the cortico-striatal-thalamic loop and tremor-dominant patients have deficits in the cerebellar-thalamic-cortical circuit (Benninger et al., 2009). However, this difference is not absolute, for the tremor-dominant PD patients tend to develop rigidity during PD progression (Levy et al., 2000). This change may be related to the progressive development of PD pathology. For example, the lesions that cause motor symptoms are initially limited to the brainstem and then gradually develop to other cortical areas (Alves et al., 2006). In general, motor symptoms initiated with rigidity and initiated unilaterally might be an important predictor of FOG.

Management of FOG usually includes drug optimization and surgical intervention, but the effects are not satisfying with every patient. Physical therapies, such as transverse bars on the floor and rhythmic auditory stimulation, are gradually being used in practice, combined with emotional therapy and cognitive improvement (Martens et al., 2014; Borcz et al., 2015; Maslivec et al., 2020). Comprehensive and systematic therapy, including medication, physical therapy, cognitive therapy, psychotherapy, and social function therapy specialized for the patient, may be the next development direction in the future.

Several limitations in this study have to be mentioned: (1) This study only recruited 64 participants, and the number of neuropsychological tests were limited. Further studies with a larger sample size and more various tests focused on striatal function are recommended to investigate FOG. (2) In our study, males were more likely to have FOG; thus, for the PD-FOG(+) and PD-FOG(−) subgroups, only the ages, years of education, and H&Y were matched. We recommend further studies to match sex as well.

## Conclusion

In conclusion, PD patients show poorer cognitive function, emotional condition, and neuropsychiatric symptoms compared with HC. When controlling for ages, years of education, and H&Y, there were no statistical differences in cognitive function between PD-FOG(+) and PD-FOG(−) patients, indicating traditional neuropsychological testing may not be sensitive enough to detect cognitive impairment in PD. Neuroimaging combined with more targeted neuropsychological tests for striatal function might be the future direction. But PD-FOG(+) patients had worse motor and non-motor symptoms than PD-FOG(−) patients. Motor symptoms initiated with rigidity and initiated unilaterally might be an important predictor of FOG.

## Data Availability Statement

The raw data supporting the conclusions of this article will be made available by the authors, without undue reservation.

## Ethics Statement

The studies involving human participants were reviewed and approved by the Medical Ethics Committee of Shanghai TongRen Hospital (ID: 2020-084-01). The patients/participants provided their written informed consent to participate in this study.

## Author Contributions

RT designed this study, did all tests, wrote this manuscript and would revise. X-PW designed this study, reviewed the manuscript, offered fund and would revise. Both authors contributed to the article and approved the submitted version.

## Conflict of Interest

The authors declare that the research was conducted in the absence of any commercial or financial relationships that could be construed as a potential conflict of interest.

## Abbreviations

ADL: Activities of Daily Living
AVLT: Auditory Verbal Learning Test
BMI: body mass index
BVMT-R: Brief Visuospatial Memory Test-Revised
CDT: Clock Drawing Test
CI: cognitive impairment
DST: Digital Span Test
EERT: Eye Expression Recognition Test
FOG: freezing of gait
FOG-Q: Freezing of Gait Questionnaire
H & Y: Hoehn and Yahr score
HAMA: Hamilton Anxiety Scale
HAMD: Hamilton Depression Scale
JLO: Judgment of Line Orientation
LED: levodopa equivalent dose
MDS-UPDRS-III: part III of the Movement Disorders Society Unified Parkinson’s disease rating scale
MMSE: Mini-Mental State Examination
MoCA: Montreal Cognitive Assessment
NMSS: the Non-Motor Symptoms Scale
NPI-Q: Neuropsychiatric Inventory Questionnaire
PD: Parkinson’s disease
PDSS: the Parkinson’s Disease Sleep Scale
RBD: REM behavior disorder
ReHo: regional homogeneity
RLS: Restless leg syndrome
SCWT: Stroop Color-Word Test
SDMT: Symbol Digit Modalities Test
SMA: supplementary motor area
SNc: the substantia nigra pars compacta
SRTT: the serial reaction time task
TMT: Trail Making Test
VFT: Verbal Fluency Test.
